# Differential Impact of Cysteine Cathepsins on Genetic Mouse Models of *De novo* Carcinogenesis: Cathepsin B as Emerging Therapeutic Target

**DOI:** 10.3389/fphar.2012.00133

**Published:** 2012-07-11

**Authors:** Thomas Reinheckel, Christoph Peters, Achim Krüger, Boris Turk, Olga Vasiljeva

**Affiliations:** ^1^Institute of Molecular Medicine and Cell Research, Albert-Ludwigs-University FreiburgFreiburg, Germany; ^2^Centre for Biological Signalling Studies, Albert-Ludwigs-University FreiburgFreiburg, Germany; ^3^Klinikum Rechts der Isar der Technischen Universität München, Institut für Experimentelle Onkologie und TherapieforschungMünchen, Germany; ^4^Department of Biochemistry and Molecular and Structural Biology, Jozef-Stefan-InstituteLjubljana, Slovenia; ^5^Faculty of Chemistry, University of LjubljanaLjubljana, Slovenia; ^6^Center of Excellence CIPKEBIPLjubljana, Slovenia; ^7^Center of Excellence Nanosciences and NanotechnologyLjubljana, Slovenia; ^8^CytomX Therapeutics Inc.South San Francisco, CA, USA

**Keywords:** cancer, cathepsin, metastasis, microenvironment, mouse model, preclinical model, protease, protease inhibitor

## Abstract

Lysosomal cysteine cathepsins belong to a family of 11 human proteolytic enzymes. Some of them correlate with progression in a variety of cancers and therefore are considered as potential therapeutic targets. Until recently, the contribution of individual cathepsins to tumorigenesis and tumor progression remained unknown. By crossing various types of mouse cancer models with mice where specific cathepsins have been ablated, we contributed to this gap of knowledge and will summarize the results in this report. The employed models are the Rip1-Tag2 model for pancreatic neuroendocrine tumors, the K14-HPV16 model for squamous skin and cervical cancers, and the MMTV-PyMT model for metastasizing breast cancer, the KPC model for pancreatic ductal adenocarcinoma, and the APC^min^ mice developing early stages of intestinal neoplasia. All models harbor mutations in relevant tumor suppressors and/or cell-type specific expression of potent oncogenes, which initiate *de novo* carcinogenesis in the targeted tissues. In all these models deletion of cathepsin B led to suppression of the aggressiveness of the respective cancer phenotype. Cathepsin B is networking with other proteases as it was shown for cathepsin X/Z. In contrast, deletion of cathepsin L was beneficial in the RiP1-Tag2 model, but enhanced tumorigenesis in the APC^min^, and the K14-HPV16 mice. A logical consequence of these results would be to further pursue selective inhibition of cathepsin B. Moreover, it became clear that cathepsins B and S derived from cells of the tumor microenvironment support cancer growth. Strikingly, delivery of broad spectrum cysteine cathepsin inhibitors in the tumor microenvironment disrupts the permissive ecosystem of the cancer and results in impaired growth or even in regression of the tumor. In addition, combination of cysteine cathepsin inhibition and standard chemotherapy improves the therapeutic response of the latter. Taken together, the next preclinical challenges for developing cathepsin inhibition as cancer therapy might be the improvement of inhibitor selectivity and targeted delivery to the tumor microenvironment and investigation of the biological context of the individual factors within the complex proteolytic network.

## Introduction

Cysteine cathepsins are papain-like peptidases of which cathepsin B, C, F, H, L, K, O, S, V, W, X/Z have been identified in the human genome and are well defined in molecular, biochemical, and structural terms (for review, Turk et al., 2001, 2012b). Cysteine cathepsins are mainly localized in the acidic cellular compartments, and are involved in numerous cell biological processes executed by the endosomal/lysosomal compartment (for review, Muller et al., 2012). A major task for the cathepsins is their involvement in the MHC class II antigen presentation pathway as well as in the cross-presentation of antigens to MHC class complexes (for review, Watts, 2012). However, cathepsins were also shown to be able to escape the acidic vesicles and mediate cell death-processes and also execute specific functions in the nucleus (for review, Turk and Turk, 2009; Reiser et al., 2010; Repnik et al., 2012). In human cancers, cysteine cathepsins are frequently overexpressed and are even secreted due to gene amplification, transcriptional activation, alternative splicing, or further posttranslational processes (for review, Mohamed and Sloane, 2006). Increased levels of cysteine cathepsins, among which cathepsins B and L received most attention, are often – but not always – correlated to a poor survival of the cancer patients (for review, Jedeszko and Sloane, 2004). Such correlations have been established for many solid tumor entities, i.e., for cancers of the adrenal, bladder, breast, cervix, colon, kidney, lung, ovary, pancreas, prostate, testis, and the thyroid grand (Pietras et al., 1979; Chauhan et al., 1991; Harbeck et al., 2000, 2001; Niedergethmann et al., 2004; Troy et al., 2004; Werle et al., 2004; Tedelind et al., 2010; Nouh et al., 2011). In general, the tumor and metastasis-promoting effect of secreted cysteine cathepsins is thought to be caused by their ability to degrade extracellular matrix molecules, which in turn enables cancer cells to invade into the surrounding tissue and to metastasize (Rothberg et al., 2012). This simplistic model is not the whole story as in some instances cysteine cathepsins can even promote tumor cell apoptosis, which would be beneficial for the patient (Vasiljeva and Turk, 2008). In addition there is evidence that proteases promote tumor growth by processing growth factors, cytokines, and chemokines or increasing their bioavailability by releasing them from the extracellular matrix (Van Damme et al., 2004; Green and Lund, 2005; Joyce and Pollard, 2009) and are therefore functionally embedded in the complex proteolytic and cellular network (Lopez-Otin and Hunter, 2010; Mason and Joyce, 2011; Turk et al., 2012a), which renders interference with proteases more difficult (Lopez-Otin and Overall, 2002; Noel et al., 2008; Kruger et al., 2010). The proteases contributing to these processes and especially the relative contribution of individual cysteine cathepsins are still subject to intense investigation.

## Genetic Mouse Models of Human Cancers

Over the past decade, considerable effort was put into studying the pathophysiological roles of individual cathepsins in complex murine models of cancer. In the first instance, these were models in which human or mouse cancer cells were injected into immunodeficient or syngenic recipient mice, respectively. The status of cathepsin expression in the tumor cells or on the recipient site has been modulated by RNA interference, knock-out technology, or overexpression (Berchem et al., 2002; Gondi et al., 2004; Lakka et al., 2004; Li et al., 2005; Alvarez-Diaz et al., 2009; Burden et al., 2009; Ward et al., 2010; Malla et al., 2011; Withana et al., 2012). In brief, these models provided solid evidence for amelioration of malignant cell behavior by inhibition of one or even more proteases in established cancer cell lines.

The focus of this review are studies, in which cathepsin knock-out or transgenic mice with overexpression of cathepsins have been crossed with transgenic mouse models of *de novo* carcinogenesis, giving the advantage of a natural co-evolution of the growing tumor and its microenvironment. These models are the seminal Rip1-Tag2 model for pancreatic neuroendocrine neoplasia (Hanahan, 1985; Folkman et al., 1989), the K14-HPV16 model for squamous skin and cervical cancers (Arbeit et al., 1994; Coussens et al., 1996), and the MMTV-PyMT model for metastasizing breast cancer (Guy et al., 1992). All three cancer models have in common, that cell-type specific promoters induce strong expression of potent viral oncogenes initiating malignant transformation and stepwise tumor progression through the distinct stages of cancer from premalignant lesions to invasive and metastasizing carcinomas. Hence, these models allow the assessment of the various cell biological aspects of carcinogenesis such as proliferation, cell death, angiogenesis, invasion, and metastasis. More recent mouse models harbor mutations critically relevant for specific types of human cancers. Mice that express a truncated Adenomatous Polyposis Coli gene product (APC^min^ and APC^Δ468^) have been used to study the role of cathepsins in early stages of intestinal neoplasia (Moser et al., 1990; Boudreau et al., 2007; Gounaris et al., 2008). The KPC mouse model, expressing mutations in the endogenous KRAS and p53 genes in the exocrine pancreas, is a faithful model of metastasizing pancreatic adenocarcinoma (Hingorani et al., 2003, 2005; Olive et al., 2004), which has been crossed to cathepsin B deficient mice (Gopinathan et al., 2012). Here we present key observations learned from investigating cathepsin-deficient mice crossed with the above-mentioned cancer mouse models and discuss cathepsin-directed therapy studies in these models.

## Cathepsins in Cancer Growth and Metastasis

Accumulating clinical and experimental data indicate that cathepsin B is a cancer-promoting protease (Poole et al., 1978; Sloane et al., 1981, 1986; Nouh et al., 2011). This concept was further supported in the Rip1-Tag2 as well as in MMTV-PyMT mice, as deficiency for cathepsin B resulted in slower cancer progression and reduced invasion (Gocheva et al., 2006; Vasiljeva et al., 2006, 2008) in both cancer models. Interestingly, the embedding of cathepsin B in the proteolytic network was documented, as a redistribution of cathepsin X/Z to the surface of cathepsin B deficient PyMT cancer cells has been detected (Vasiljeva et al., 2006). Hence, cathepsin Z was suspected to compensate for the loss of cathepsin B, a view supported by the fact that cathepsin B and cathepsin Z are the only enzymes with carboxypeptidase activity among the cysteine cathepsins (Klemencic et al., 2000). Indeed, analysis of cathepsin B/Z double-deficient mice in the context of the MMTV-PyMT breast cancer model revealed a strongly reduced tumor and lung metastatic burden, while a single cathepsin Z deficiency had no clear effect on the overall tumor phenotype (Sevenich et al., 2010). In order to model the situation of human cancers, which often show high cathepsin B expression, transgenic mice overexpressing human cathepsin B were crossed with MMTV-PyMT mice (Sevenich et al., 2011). These mice showed significantly higher tumor burden and increased lung metastasis, which further supports a tumor-promoting role of high cathepsin B levels. In accordance, cathepsin B deficiency in APC^min^ mice reduced formation of intestinal neoplasia (Gounaris et al., 2008). Taken together, investigation of cathepsin B deficiency and cathepsin B overexpression in four unrelated genetic mouse models of *de novo* tumorigenesis showed consistently that cathepsin B is a tumor-promoting protease and, therefore, a potential therapeutic target.

Single deficiencies for cathepsins B, L, S, and H perturbed the development of Rip1-Tag2 pancreatic islet cancers, while deletion of cathepsin C did not affect tumor progression in this model (Gocheva et al., 2006, 2010a; Wang et al., 2006). Cathepsin L-ablated mice showed increased carcinogenesis and frequency of lymph node metastasis in K14-HPV16 skin cancer mice (Dennemarker et al., 2010). Interestingly, crossing of *furless* mice, which harbor a spontaneous active site mutation of cathepsin L, with APC^min^ mice increased the multiplicity of premalignant intestinal polyps significantly (Boudreau et al., 2007). Hence, the contradictory result of cathepsin L ablation in the Rip1-Tag2 as compared to the APC^min^ and K14-HPV16 models is an impressive example for the context-dependent consequences of a gene knock-out (Kruger, 2009). The reason for context-specific effects of individual cathepsins in tumors might well be the fact that these proteases play contrasting roles in carcinogenesis by acting as positive mediators of invasion (pro-tumor), as positive or negative regulators of proliferation (pro- or anti-tumor effect), and, in addition, may affect cell death (Goulet et al., 2004; Vasiljeva and Turk, 2008; Vasiljeva et al., 2008; Turk and Turk, 2009). The relative importance of these tumor biological processes in a given cancer entity will determine the positive or negative effect of a genetic or pharmacologic modulation of cathepsin activity in the context of an individual malignancy.

## Cathepsins in Tumor-Associated Inflammation and Angiogenesis

The active roles of the tumor microenvironment in carcinogenesis and tumor progression have attracted considerable attention during the past decade. It emerged that tumor-promoting inflammation and induction of angiogenesis are hallmark characteristics of activated tumor stroma (for review, Hanahan and Weinberg, 2011). The genetic cancer mouse models discussed here have also been instrumental to define the role of cathepsins and endogenous cathepsin inhibitors in the tumor microenvironment. Early studies of intravenous injection of cancer cells isolated from MMTV-PyMT mice into congenic cathepsin B deficient mice revealed a decreased number and size of the resulting lung colonies as compared to wild-type recipient mice (Vasiljeva et al., 2006). Macrophages closely associated with these experimental lung metastases show elevated expression of cathepsin B, suggesting that cathepsin B expressed by metastasis-associated macrophages has a pro-metastatic effect (Vasiljeva et al., 2006). The tumor-promoting role of cathepsins in tumor-associated immune cells has been elegantly proven by transfer of cathepsin-deficient bone marrow into the Rip1-Tag2 model of pancreatic neuroendocrine neoplasia (Gocheva et al., 2010b). The experiment revealed that cathepsins B and S in innate immune cells promote indeed cancer progression while cathepsins C and L did not induce this effect (Gocheva et al., 2010b). Transfer of cathepsin L-deficient bone marrow into the K14-HPV16 mouse model of skin cancer did also not affect cancer progression (Dennemarker et al., 2010). However, in both cancer models tumor progression is significantly altered in cathepsin L knock-out mice receiving wild-type bone marrow, suggesting a role for tumor cell-derived cathepsin L or cathepsin L expressed by non-myeloid cells of the tumor stroma. The importance of cathepsins in the tumor microenvironment is further supported by the finding, that genetic ablation of the extracellular cysteine cathepsin inhibitor cystatin C facilitates the development of premalignant dysplasia in the epidermis of K14-HPV16 mice (Yu et al., 2010). Tumor-associated inflammation is interconnected with angiogenesis induction within the tumor. Tumorigenesis in Rip1-Tag2 mice is highly dependent on massive induction of blood vessel formation – the so called “angiogenic switch.” Ablation of cathepsins B, L, S, and H, but not of cathepsin C, resulted in impaired vascularization of the pancreatic tumors (Gocheva et al., 2006, 2010a; Wang et al., 2006). Evidence for a functional role of cathepsins in other, less angiogenesis-driven, cancer models is slowly emerging. Overexpression of human cathepsin B in MMTV-PyMT mice resulted in increased vascular density within the breast cancers (Sevenich et al., 2011) and anti-angiogenic efficacy of an antibody targeting cathepsin S was shown in xenograft models (Ward et al., 2010).

## Cathepsins as Targets for Cancer Therapy

Overexpression and extracellular re-dislocation of cysteine cathepsins have been associated with multiple stages of tumorigenesis and tumor progression (Vasiljeva et al., 2007). Thus, inhibition of cysteine cathepsins could be a potent strategy for treating cancer. The first study on the use of the broad-spectrum small molecule cysteine cathepsin inhibitor, JPM-OEt, in the Rip1-Tag2 pancreatic islet cells cancer mouse model demonstrated significant anti-tumor efficacy in three distinctive trial designs: prevention, intervention, and regression (Joyce et al., 2004), that target different stages in tumorigenesis (Bergers et al., 1999). Furthermore, combination of cathepsin inhibition with two distinct regimens of chemotherapy administration (MTD or chemo-switch) was shown to lead to a more pronounced tumor regression, decreased tumor invasiveness, and increased survival in the Rip1-Tag2 model (Bell-McGuinn et al., 2007). However, there might be some limitations for the use of small synthetic probes in the clinic, primarily because of their pharmacokinetic properties, such as relatively short circulation half-life and poor bioavailability, due to the rapid conversion of the injected cell permeable ethyl esters, e.g., JPM-OEt, to its corresponding acid in the serum (Sadaghiani et al., 2007). The latter, most probably appeared to be the reason for the failure of the JPM-OEt inhibitor of cysteine cathepsins in the treatment study with the MMTV-PyMT transgenic mouse mammary cancer model performed in two trials on early and advanced cancers (Schurigt et al., 2008). Notably, orthotropic injection of murine 4T1.2 mammary cancer cells into the mammary gland fat pad and subsequent systemic administration of the highly selective cathepsin B inhibitor CA-074 reduced bone metastasis, while JPM-OET did not (Withana et al., 2012). Interestingly, this anti-metastatic effect was not found for application of the widely used broad spectrum inhibitor JPM-OET, highlighting the need for selective inhibition of cathepsin B.

Despite the important role of tumor cell-derived cathepsins in cancer progression, there is an increasing body of evidence confirming the up-regulation of cysteine cathepsins by macrophages in the tumor microenvironment, such as macrophages (Mohamed and Sloane, 2006). Notably, overexpression of cathepsins by macrophages has been demonstrated to be induced by a direct interaction with tumor cells (Vasiljeva et al., 2006), by interleukins (Gocheva et al., 2010b), or by chemotherapy (Shree et al., 2011). Moreover, the study of Shree et al. demonstrated a massive attraction of macrophages to tumor sites treated with chemotherapy, with consecutive up-regulation of cathepsins by those cells. Interestingly, secreted cathepsins were shown to induce tumor cell resistance to the chemotherapy that has been supported through co-culture and treatment experiments (Shree et al., 2011). Thus, the approach of targeting macrophage-derived cathepsins could be, on one hand, beneficial for the inhibition of tumor progression and invasion, and, on the other hand, could induce tumor cells’ sensitivity to the death signals and will increase efficacy of standard chemotherapy drugs. In that respect, novel technologies enabling targeting of cells of the tumor microenvironment would be very potent in terms of the targeted delivery of cysteine cathepsin inhibitors to the cells of tumors primarily overexpressing cathepsins (e.g., macrophages) and would improve pharmacokinetic properties of small synthetic drugs by encapsulation in nano-carriers (Mikhaylov and Vasiljeva, 2011). Moreover, such a system for targeted drug delivery based on magnetic nanoparticles and biocompatible lipid shell, forming ferri-liposomes, has been recently developed and validated for cathepsin inhibition by the JPM-OEt inhibitor in an orthotopically transplanted mammary mouse cancer model (Mikhaylov et al., 2011). Notably, targeted delivery and increased bioavailability of inhibitor through the use of ferri-liposome nano-carriers resulted in significant cathepsin inhibition in distant organs and led to significant reduction of mammary tumor burden volume that have been confirmed by the alterations in tumor marker expression, as Ki67 and E-cadherin (Mikhaylov et al., 2011).

Taken together, the accumulated evidence in the literature strongly supports the concept of the use of cathepsins, in particular cathepsin B, as targets for cancer therapy targets. Therefore, inhibitors with improved pharmacokinetic properties and improved selectivity, possibly combined with appropriate delivery systems, and their use in combination with established chemotherapeutic treatment strategies, will have the potential to become valuable therapeutics for the treatment of metastatic malignant disease in the clinic.

## Conflict of Interest Statement

The authors declare that the research was conducted in the absence of any commercial or financial relationships that could be construed as a potential conflict of interest.
